# Supplementary material for: The Sex-Dependent Ameliorative Effect of Intermittent Fasting on Urinary System Functions in Genetic Absence Epileptic Rats
Source: Biology (Basel). 2025 Feb 4;14(2):158. doi: 10.3390/biology14020158 (PMC11852256; doi:10.3390/biology14020158)
Supplement: Supplementary file 1 [file biology-14-00158-s001.zip › biology-3360523-supplementary.pdf]

	Wistar Control	Wistar + ADF	GAERS	GAERS + ADF
LogEC50	-6,3931	-6,3381	-6,3324	-6,3583
EC50	4,045 e-007	4,591 e-007	4,652 e-007	4,382e-007

Table S1. LogEC 50 and EC50 values for the contractile responses obtained from the cumulative dose-response curves to CCh of female bladder tissues (n=4-6 for each group).
